# Investigation of the Structural Changes in Silk Due to Tin Weighting

**DOI:** 10.3390/polym16172481

**Published:** 2024-08-30

**Authors:** Ibrahim Elrefaey, Hend Mahgoub, Chiara Vettorazzo, Marjan Marinšek, Anton Meden, Andrej Jamnik, Matija Tomšič, Matija Strlič

**Affiliations:** 1 Faculty of Chemistry and Chemical Technology, University of Ljubljana, Večna Pot 113, 1000 Ljubljana, Slovenia; 2 ARCHES Research Group, University of Antwerp, Blindestraat 9, 2000 Antwerp, Belgium; 3 AXIS Research Group, University of Antwerp, Groenenborgerlaan 171, 2020 Antwerp, Belgium

**Keywords:** silk, tin-weighting, structural changes, X-ray analysis, IR spectroscopy

## Abstract

In the 19th century, the weighting of silk with metal salts, such as tin, was a common practice to enhance certain properties of silk fabrics and compensate for the weight loss incurred during the degumming process. This technique induces both physical and chemical modifications to the fibres, contributing to their long-term degradation, which requires thorough investigation. This study aims to examine the structural changes in silk fibres caused by the accumulation of metal salts from the tin-weighting process, using mock-up samples prepared through successive loading with weighting agents using a traditional tin-phosphate treatment method. Unweighted and tin-weighted silk samples were compared using scanning electron (SEM) micrographs, which presented the dispersed nanoparticles on the fibres, while through energy-dispersive X-ray spectroscopy (EDS) elemental mapping, the presence and uniform distribution of the weighting agents were confirmed. Fourier-transform infrared spectroscopy (FTIR) analysis revealed structural changes in tin-weighted silk samples compared to untreated ones, including shifts in amide bands, altered water/hydroxyl and skeletal stretching regions, and increased skeletal band intensities suggesting modifications in hydrogen bonding, β-sheet content, and structural disorder without significantly impacting the overall crystallinity index. X-ray diffraction (XRD) analysis of both pristine and tin-weighted silk samples revealed significant alterations, predominantly in the amorphous regions of the silk upon weighting. These structural changes were further examined using small-angle X-ray scattering (SAXS) and small- and wide-angle X-ray scattering (SWAXS), which provided detailed insights into modifications occurring at the nanometre scale. The analyses suggested disruptions in β-sheet crystals and intermolecular packing, especially in the amorphous regions, with increasing amounts of tin-weighting. Contact angle analysis (CA) revealed that the tin-phosphate-weighting process significantly impacted silk surface properties, transforming it from moderately hydrophobic to highly hydrophilic. These changes indicate that the incorporation of tin-phosphate nanoparticles on and within silk fibres could restrict the flexibility of polymer chains, impacting the physical properties and potentially the degradation behaviour of silk textiles. By studying these structural changes, we aim to deepen our understanding of how tin-weighting impacts silk fibre structure, contributing valuable insights into the longevity, conservation, and preservation strategies of silk textiles in the context of cultural heritage.

## 1. Introduction

For millennia, silk has captivated humanity with its softness, elegant drape, and luminous sheen. This remarkably versatile material has been a cornerstone of textile production [1,2], incorporated in everything from delicate garments to magnificent tapestries [3]. The general composition of raw silk is 72–81% fibroin, 19–28% sericin, 0.8–1.0% fat and wax, and 1.0–1.4% colouring matter and ash [1]. The composition differs depending on the species of silkworm and their diet, environmental and genetic factors, and processing techniques. Silk’s exceptional properties are due to its unique makeup: the fibrous protein Silk Fibroin (SF) typically comprises of a light chain and a heavy chain, linked by disulfide bonds to form an H-L dimer, often associated with a glycoprotein. The H-chain, the primary component, is rich in glycine, alanine, and serine, though proportions vary among species. The SF structure features alternating crystalline and amorphous domains. Crystalline regions contain repetitive sequences of small amino acids, while amorphous areas have diverse residues. Semi-crystalline sections often include aromatic or hydrophobic residues. This structural arrangement gives silk its characteristic strength, elasticity, and biocompatibility, with specific properties varying among silkworm species. The combination of these structural elements determines the unique qualities of silkworm silk [4].

Transforming raw silk into luxurious textiles requires several processing steps. Among these are degumming and bleaching, which remove the natural sericin coating, along with pigments and impurities. This process, while achieving silk’s softness and lustre [1], also reduces its weight. While the weight loss is affected by many degumming process factors, it can represent a quarter of its raw weight [5,6,7]. Other steps, such as weighting and dyeing are used to further enhance versatility, expanding the range of colours, weights, and textures available to artisans [1].

The 19th-century practice of loading silk with metal salt solutions most often involved tin chlorides. Originally used to compensate for the weight lost during degumming and to improve drape, it also introduced potential problems. As silk transitioned from being sold by length to weight, the desire for increased weight could lead to attempts to inflate weight at the expense of fibre integrity. While moderate weighting could improve drape and dyeability, excessive use could compromise the long-term condition of the silk fibre [8]. Generally, the tin-weighting technique using a stannic chloride, SnCl_4_, solution results in a gel or a precipitate of stannous (tin(II)) hydroxide Sn(OH)_2_, or stannic (tin(IV)) oxide SnO_2_ that penetrates the pores of the fibres, and hydrochloric acid which is washed away in the washing step. The disodium phosphate Na_2_HPO_4_ bath is used to fix tin hydroxide and neutralize the hydrochloric acid. In this basic tin-weighting process the weight gain is approximately 8% per weighting round or cycle [9]. 

Understanding how weighting affects the silk structure is crucial for its preservation, as the amount of metal loading could impact the extent of potential damage [10]. Despite the unique physical properties of silk, silk textiles tend to be susceptible to deterioration when exposed to environmental stressors like light, heat, and humidity, and even the weighting techniques used in their creation [6,11,12,13]. 

Characterisation of fibrous protein polymers such as silk presents a longstanding challenge in the understanding of their chemical and structural degradation. Understanding how the structure changes upon weighting is critical for optimizing silk performance and developing preservation strategies. Silk has been studied using a large number of techniques to examine its composition and structure [14], including microscopic, spectroscopic, and chromatographic methods. Microscopic investigation helped in the identification of species of silkworm, morphology and structure of silkworm cocoons, and degradation signs such as cracking of degraded silk [15,16]. FTIR spectroscopy serves multiple purposes such as the monitoring of protein conformation, examination of degradation, and identification of degradation markers [17,18,19,20]. Chromatographic analysis such as size exclusion chromatography (SEC) has been used to determine molecular weight distribution, which has enabled researchers to better understand the changes in molar mass distribution that occur as a result of the treatment or degradation of silk fibroin [21,22,23]. X-ray diffraction/scattering has been employed in many investigations of silk, including probing nano-scale structures [24,25] assembly [26], and degradation [27,28,29]. Nevertheless, there is still much to be explored, especially in establishing a clear relationship between the chemical and physical properties of silk fibres during degradation.

This study adopts an experimental approach using modern silk samples to investigate the influence of metal salt weighting on silk fabric structure. Samples with systematic variations in tin-weighting levels are prepared to assess structural changes. The morphological, elemental, and structural characterization of pristine and weighted silk samples is conducted using SEM-EDS, FTIR-ATR, X-ray diffraction (XRD), and small-angle and wide-angle X-ray scattering (SAXS/WAXS), in addition to Contact Angle measurements (CA). The findings of this research aim to contribute to the understanding of the effect of tin weighting on the structure of silk. 

## 2. Material and Methods

### 2.1. Sample Preparation

A set of tin-weighted silk fabric mock-ups were prepared from plain-weave Fuji silk (73 g/m^2^) purchased from IDEEN, Düsseldorf, Germany. The tin-weighted samples were prepared following a standardised method derived from historical tin-weighting techniques [9,11,12,30,31].

The silk was first washed in warm deionised water (30 °C, soaked for 1 h) and hung to dry in air. Once dry, the starting weight of the silk was recorded. Around 0.4 m^2^ of silk is submerged in enough aqueous SnCl_4_ weighting solution to fully submerge all the silk pieces at room temperature for 4 h with regular stirring. The SnCl_4_ solution was prepared at a density of 1.210 g/mL (equivalent to 25 °Bé (Baumé)) from SnCl_4_·5H_2_O purchased from Thermo Fisher Scientific (ThermoFisher (Kandel) GmbH), Kandel, Germany). Samples were then removed from the bath and rinsed thoroughly in running deionised water for 1 min before being placed in an aqueous disodium phosphate bath (prepared at 1.0507 g/mL, equivalent to 7 °Bé, from Na_2_HPO_4_ purchased from Carl Roth, Karlsruhe, Germany) at room temperature. After 1 h, the silk was removed, rinsed in deionised water for 1 min and hung to air dry. Samples were then weighted, and the weight increase noted. Immersion in the stannic chloride and disodium phosphate baths was repeated to obtain samples treated 1, 2, 3 and 4 times (marked as 1P, 2P, 3P and 4P, respectively). Washed, but not weighted samples (NW) from the same fabric were used for comparison in this study.

The successive repetition of the treatment was aimed at exploring the cumulative effects of metal salt incorporation on silk fabrics, as well as being in line with the historical practices reported in the literature. The weight gains differ according to the recipe, method and condition of the weighting process [30]. 

### 2.2. Analytical Techniques

#### 2.2.1. SEM-EDX Analysis

For the fibre morphology and elemental analysis, a Zeiss ULTRA plus scanning field emission electron microscope (FE-SEM) from Jena, Germany was employed. This instrument is equipped with a Schottky field emission system and an in-lens secondary electron detector (SE) placed above the objective lens. Energy-dispersive X-ray spectroscopy (EDX) analysis was facilitated by an Oxford X-Max SDD detector, sourced from the Oxford, United Kingdom, with a working surface area of 50 mm^2^, coupled with the INCA 4.14 X-ray microanalysis software package for data processing.

Sample preparation involved the fixation of a 3 mm^2^ silk fabric sample on conductive carbon tape, and fixed on the pin stubs, followed by sputter-coating with a thin layer of gold/palladium (Au/Pd) to enhance conductivity. The SEM analysis was performed using a magnification range of 20,000 to 100,000 times (20.00 to 100.00 KX), a spot size of 0.5000 μm, an accelerating voltage of 2.00 kV, a field of view of 30.00 μm, and a working distance of 4.5 mm. The estimation of nanofeature size distribution was conducted from SEM micrographs. Image size scales were calibrated, and the diameters of individual nanofeatures on the fibre surface were manually measured using ImageJ 1.54g software. The average nanofeature size was determined by fitting the particle size distribution histogram to a log-normal distribution function in OriginLab Pro 2024 software [32].

#### 2.2.2. FTIR-ATR Analysis

FTIR-ATR measurements were performed using a Bruker FTIR Alpha II spectrometer equipped with an ATR platinum Diamond 1 accessory. The instrument parameters were set with a resolution of 4 cm^−1^, sample scan time of 64 scans, background scan time of 64 scans, spectral range of 400 to 4000 cm^−1^, and the measurement mode was set to Absorbance. A background spectrum was collected before each sample measurement to compensate for atmospheric influences. The fabric samples were carefully positioned on the ATR crystal, ensuring that the fabric direction was maintained consistently between different samples. The instrument’s pressure knob was used to apply nearly consistent pressure on the samples during the measurements. The scanning and processing of the spectra were performed using the OPUS 8.7 software. Spectra were recorded in the specified spectral range, and the acquired data were processed using OriginLab Pro 2024 for baseline correction and peak analysis. The Deconvolution of the amide bands was performed to differentiate vibrational modes associated with various secondary structures (β-sheet, α-helix, random coil) based on the established literature and studies [18].

#### 2.2.3. XRD Analysis

X-ray diffraction (XRD) of the samples was collected at room temperature with a PANalytical X’Pert PRO high-resolution powder diffractometer using Cu-K_α1_ radiation (λ = 1.5406 Å) in the reflection mode. The silk fabric samples (1 × 2 cm^2^) were directly placed and stretched over a silicon plate sample holder “zero-diffraction plate”. An automatic divergence slit and a horizontal mask were used so that a sample area of 10 × 10 mm^2^ was irradiated throughout the scanning. Data were collected in the 2*θ* range from 5 to 53° in steps of 0.033° using a 128-channel linear multi-strip detector to achieve a total integration time of 1000 s per step. Data fitting was conducted using OriginLab Pro 2024 software, employing Multi-Peak Fitting with Gaussian functions.

#### 2.2.4. SAXS and SWAXS Analysis

The X-Ray Scattering experiments were conducted using an in-lab-modified Kratky-type camera (Anton Paar KG, Graz, Austria) equipped with a conventional X-ray generator (GE Inspection Technologies, Seifert Isodebyeflex 3003, Lewistown PA, USA) with a Cu-anode operating at 40 kV and 50 mA (Cu-Kα line with the wavelength λ = 1.54 Å) [33]. The camera was configured with a focusing multilayer optics (Göbel mirror) and block-collimation unit providing a well-defined, line-collimated primary beam. 

The silk samples (0.8 × 2.2 cm^2^) were placed directly into the evacuated camera and measured. SWAXS measurements were captured on a 2D-imaging plate, which was exposed to the scattered X-rays for 10 min and read off afterwards utilizing an imaging-plate reader (Fuji, BAS 1800II, Chiryu, Japan) with a spatial resolution of 50 × 50 μm^2^/px, but for the SAXS data, the Mythen 1K detector (Dectris, Baden, Switzerland) was used. The SAXS data were recorded in the range of the scattering vector, q, values from 0.08 to 7 nm^−1^, where q = 4π ⁄ λ ∙ sin (θ/2), with θ being the scattering angle, and the SWAXS in the range from 0.1 to 30 nm^−1^. The resulting data were still experimentally smeared due to the finite dimensions of the primary beam [34].

#### 2.2.5. Contact Angle and Hydrophilicity

Silk fabric samples were prepared for contact angle measurements to assess the impact of tin-phosphate-weighting on its hydrophilicity. The fabric was cut into pieces measuring approximately 3 cm × 1 cm. The samples were then attached to microscopic glass slides using tape to ensure a flat surface for measurement. Three silk samples were prepared: NW as a control, 1P, and 4P.

Contact angle measurements were performed using an Attension Theta optical goniometer (Biolin Scientific Oy, Espoo, Finland) with OneAttension version software (Biolin Scientific). The sessile drop method was employed to quantify wettability by measuring the static contact angle between a liquid droplet and the solid surface. Distilled water was used as the test liquid, with 5 μL droplets deposited on each sample surface. The tensiometer captured images at a rate of 2.8 frames per second, allowing for the measurement of contact angles, droplet volume, and baseline over time. Multiple measurement attempts were made for each sample type, denoted as #1, #2, and #3, to ensure reproducibility. Data collection continued for up to 70 s where possible, though rapid absorption in some samples limited the measurement duration.

## 3. Results

### 3.1. Weight Gain of Tin-Weighted Silk

Successive tin-weighting cycles led to a successive increase in fabric weight. Compared to the initial weight of the degummed silk, samples exhibited weight gains of 7.8%, 18.9%, 27.7%, and 42.2% after 1, 2, 3, and 4 weighting cycles (1P–4P), respectively. It is important to note that traditional weight gain calculations are often based on the weight of raw silk [30]; however, in this study, the initial weight of the degummed silk was used as a reference point to assess the impact of tin weighting on the treated fabric. 

### 3.2. Morphology and Elemental Mapping

**Figure 1:**
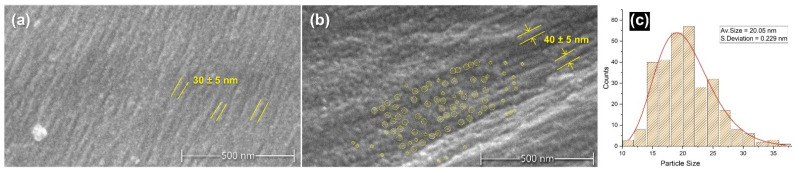
SEM micrographs of silk fibres depicting the effect of tin-phosphate weighting: (**a**) NW silk sample showing nanostructure of the surface of a silk fibre highlighted in yellow lines; (**b**) 4P silk sample showing rougher surface, surface nanostructure size modification represented by yellow lines in addition to highlighting sample area in yellow circles showing the distribution of spherical nanofeatures; (**c**) the average size of nanofeatures distribution for 4P sample fitted with a log-normal distribution function.

To understand the effect of tin weighting on the structure of the silk and the nature of the distribution of the metal salts on the fibres, SEM micrographs and EDS elemental mapping were conducted. Figure 1 presents scanning electron (SEM) micrographs captured at a magnification of 50,000 times, revealing the nanoscale morphology of silk fibres. The left image (a) displays an unmodified, NW silk fibre, showcasing its surface topography and fibrillar structure as highlighted in yellow on the fibre’s surface. Typically, an unmodified silk fibre would also exhibit other features such as residuals of sericin or some contaminants with irregular sizes. In contrast, the middle image (b) depicts a silk fibre after undergoing four successive baths of the tin-phosphate-weighting technique (4P). The image of the weighted sample exhibits bright spots, and yellow circles highlight these nanofeatures showing part of their distribution on the fibre’s surface. These bright spots are likely attributable to tin-phosphate nanoparticles deposited on the fibre surface and their effect on the swelling of the bead-like structure of fibrils during the weighting process. The scale bar in the images highlights the nanometre-scale dimensions of the fibre structure [35,36,37] in addition to the round nanofeatures, the size of which is ca. 20 nm with a standard deviation of 0.229 nm (Figure 1c). Additional examples of the samples can be found in the Supplementary Materials.

**Figure 2:**
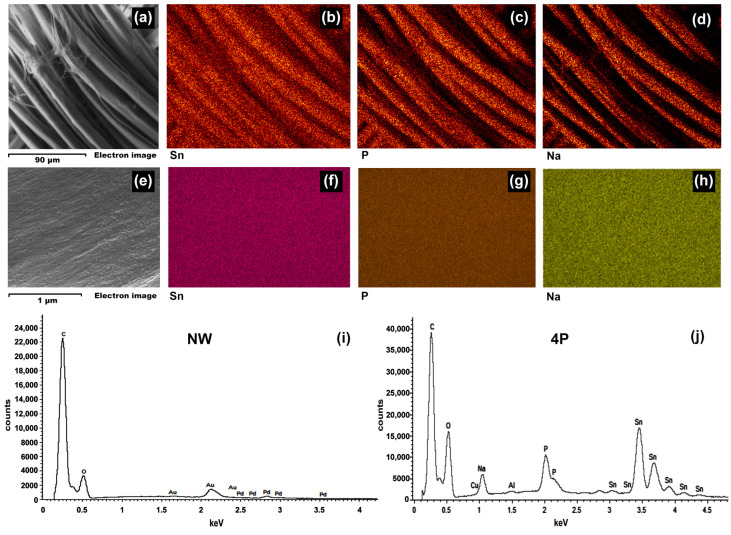
Characterization of tin-weighted silk fibres on the weighted 4P silk sample: (**a**) Low-magnification SEM micrograph (scale bar: 90 µm) showing the overall morphology of the tin-weighted 4P silk fibres. (**b**–**d**) Corresponding elemental maps illustrating the distribution of Sn, P, and Na, respectively, across the fibre surface. (**e**) High-magnification SEM micrograph (scale bar: 1 µm) focusing on a single fibre. (**f**–**h**) Elemental maps highlighting the distribution of Sn, P, and Na on the surface of an individual fibre. (**i**) EDS spectrum of the (NW) sample. (**j**) EDS spectrum of the 4P sample confirming the presence of Sn, P, and Na.

To elucidate the nature of the observed nanostructures, energy-dispersive X-ray spectroscopy (EDS) analysis was performed on NW and 4P samples, as shown in Figure 2. The EDS spectra (Figure 2i,j) for the 4P sample revealed the presence of Sn, P, and Na compared to the NW sample, indicative of the tin-phosphate-weighting process. Elemental mapping images (Figure 2b–d,f–h) confirmed a uniform distribution of these elements across both the silk fibre bundle and individual fibres, suggesting the formation of tin-phosphate nanoparticles on the fibre surface [12].

### 3.3. Structural Investigations

The physical properties of silk fibroin are largely determined by its secondary structure and hierarchical organization. The hydrophobic domains of the silk polymer chains, consisting of repeating amino acid sequences, form nano-crystalline β-sheets. These hydrophobic domains are linked by hydrophilic regions composed of bulky and polar side chains, forming the amorphous part of the secondary structure. The amorphous blocks adopt a random coil conformation, which gives elasticity to silk. These factors, the crystalline and amorphous regions, play a crucial role in determining the mechanical properties of a given silk [38].

**Figure 3:**
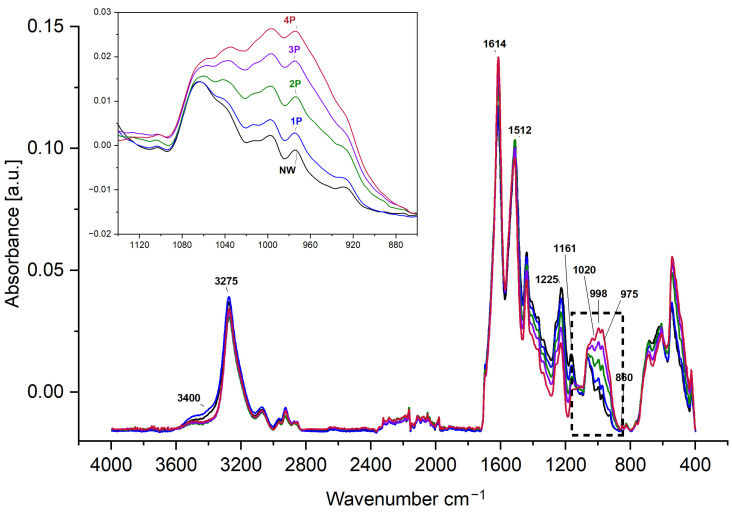
FTIR-ATR spectra for NW reference silk compared to the 1P, 2P, 3P, and 4P samples. The dashed box highlights the spectral region from 1140–860 cm^−1^, where the accumulated effect of tin-phosphate weighting is evident.

In this research, the structure of silk samples has been compared between the reference NW with its typical characteristics and conformational patterns [39] against the samples of four successive loadings of tin-weighted (1P, 2P, 3P, and 4P). For the FTIR-ATR analysis (Figure 3), the NW and 1P samples have closer or similar observations in relation to the water and hydroxyl content that appear in the broad absorption at 3400 cm^−1^ and the relatively wider peak centred at 3275 cm^−1^. The FTIR dataset is available in the Supplementary Materials. The 2P, 3P, and 4P samples show a sharper, narrower peak at 3273 cm^−1^ and lower absorption at 3400 cm^−1^. This could be attributed to the less water content or less accessibility to water molecules within the silk fibre due to the incorporation of metal salts, and the influence of tin oxide typically at around 3370 cm^−1^ [40]. Structural changes and alterations in molecular interactions induced by the weighting process could affect the hydrogen bonding N-H stretching absorption at around 3300 cm^−1^, overlapping with the O-H hydroxyl patterns [41], contributing to the observed intensity reduction. This decrease in the availability of O-H groups may influence the water absorption and, consequently, the flexibility and strength properties of the weighted silk fibres [42].

The effect of tin-phosphate weighting can be clearly observed at 1140–860 cm^−1^ and the gradual increase in peak intensities in the skeletal or carbon backbone stretching region, and, in particular, the weak band at 1015 cm^−1^ and the signature bands of silk fibroin at 998 cm^−1^ and 975 cm^−1^, which arise from the glycine–glycine and alanine–alanine sequences [12,17] These sequences generate a close network of hydrogen bonds among fibroin chains, giving rise to the typical β-sheet crystalline structure of silk. The increase in intensities could be attributed to an increase in β-sheet conformation or tighter packing induced by the weighting elements [43]. 

For a semiquantitative understanding of the alterations, a calculation of the Crystallinity Index (CI) can be obtained from the amide I or amide III region peaks. It is important to note that the crystallinity index refers to the relative β-sheet structure content [44], not true crystallinity, which can be determined using different methods either using the ratio of the absorbances at 1260 cm^−1^ (attributed to β-sheet content) and ca. 1235 cm^−1^ (attributed to random coil or the amorphous phase) [18] or the ratio of 1615 cm^−1^ (β-sheet) and 1655 cm^−1^ (α-helix and/or random coil) [27]. Another option to calculate the crystallinity index is from the peak area integration of β-sheet peaks after deconvolution [45].

**Figure 4:**
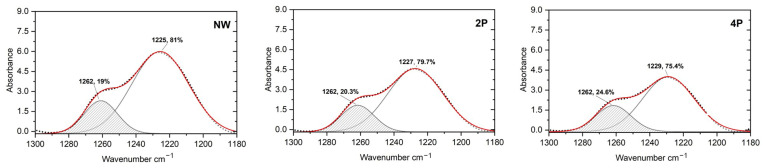
Deconvolution of FTIR spectra of amide III for β-sheet and (α-helix and/or random coil) content calculation of NW silk sample (**left**), 2P silk sample (**middle**), and 4P silk sample (**right**).

In this research, the β-sheet and α-helix and/or random coil contents were calculated from the deconvoluted peak of amide III at around 1262 cm^−1^ and 1225–1229 cm^−1^, respectively. It has been determined that there is a reduction in the random coil to an increase in the β-sheet of around 5.6% in the 4P sample compared to the NW sample, as Figure 4 shows.

**Figure 5:**
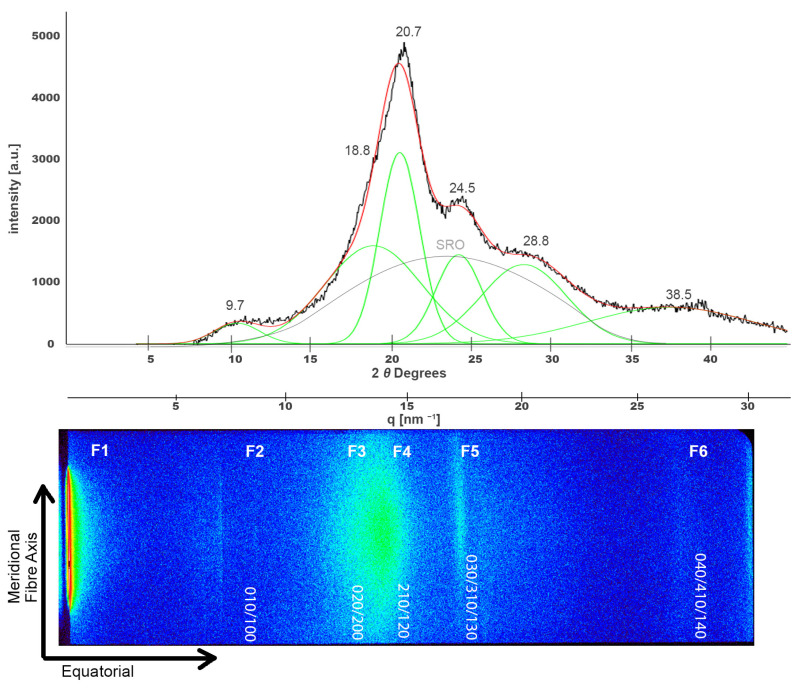
Deconvoluted XRD peaks for a pristine NW silk sample (**top**). The black line represents the original diffractogram, the red line is the fitting curve, the deconvoluted peaks are shown in green and (SRO) peak is highlighted in grey. This XRD spectrum is shown alongside the 2D SWAXS scattering image at the (**bottom**) where different scattering features are highlighted as F1 to F6.

Regarding the X-ray analysis of silk structure, samples were examined using XRD, SAXS, and SWAXS. The XRD diffractogram of pristine NW silk is expected to show a broad semi-crystalline pattern exhibiting characteristic Bragg peaks at about 2*θ* values of 9.7°, 20.7°, and 24.5° [46], corresponding to the crystalline β-sheet content and ordered packing of fibroin chains. As can be seen in the upper part of Figure 5, the diffractogram indeed shows the characteristic peaks of silk structure at around 2*θ* values of 9.7°, 18.8°, 20.5°, 24.5°, and also at 28.8° and 38.5° and the broad peak of the amorphous short-range order [24,47,48]. For the purpose of better visualization and clarity, this diffractogram was roughly aligned with the 2D image of the SWAXS result shown at the bottom that depicts characteristic intensities at (010), (020), (210), (030), and (040). In addition, different features were highlighted on the SWAXS image and will be referred to as F1, F2, F3, F4, F5, and F6. The peak on the very left side of the SWAXS image is in part the consequence of the attenuated primary beam and is used to designate the scattering angle of zero.

**Figure 6:**
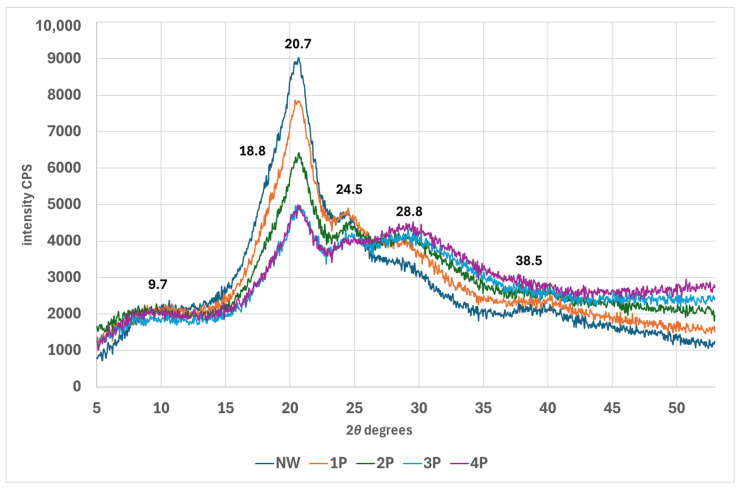
XRD diffractogram of NW and tin-weighted silk fabric.

As can be seen in Figure 6, upon metal salt weighting, peaks at 9.7°, 18.8°, 20.7°, and 24.5°, 2*θ* notably decrease in intensity, in contrast, the peak at around 29.3° related to lateral fibroin packing increases and broadens in the weighted silk. The complete XRD dataset is available in the Supplementary Materials. In X-ray diffraction (XRD) analysis of silk weighted with tin-phosphate nanoparticles, the long-range ordered crystalline structures produce distinct diffraction peaks in the diffractogram. However, short-range ordered or amorphous regions do not diffract X-rays coherently and thus do not contribute to sharp diffraction peaks. The FTIR analysis reveals an increase in β-sheet content, indicating enhanced local order within the silk fibre structure. This is supported by XRD data showing increased short-range order and an associated decrease in long-range order peaks. These findings suggest the formation of smaller, ordered domains, potentially at the expense of larger, crystalline regions. As nanoparticle concentration increases, the trend toward shorter-range order becomes more pronounced, as evidenced by a further reduction in long-range order peak intensity in the XRD pattern [43].

It has been reported that different preparation methods using tin(II) and tin(IV) yield tin phosphates exhibiting diverse crystal structures and varying oxidation states of tin [49]. As a result, amorphous tin phosphate can be produced. In the process of weighting, the tin salt (SnCl_4_) is absorbed into the silk fibres and can be hydrolysed to form tin(IV) hydroxide species. These then react with the phosphate anions from Na_2_HPO_4_ to form insoluble tin(IV)-phosphate nanoparticles or crystallites dispersed within the amorphous regions of the silk fibroin structure [12]. This contributes to the broad Bragg peaks presented in weighted silk. The increase in the spectra at the high-angle region that starts from 40° can also be attributed to the broad peak of tin(IV) phosphate [49]. The abundant tin(IV)-phosphate nanoparticles promote a broad diffraction peak centred at around 29°, and it seems that the scattering from the tin(IV) phosphate dominates over the original silk peaks, effectively obscuring features like the β-sheet crystal peaks. 

**Figure 7:**
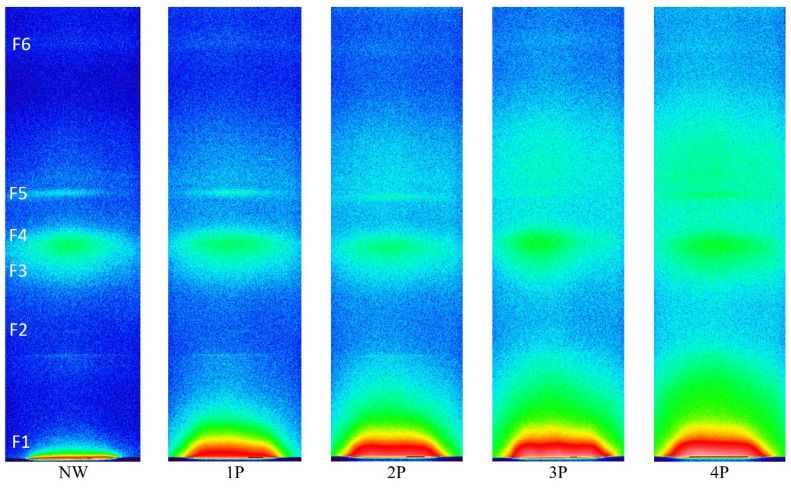
SWAXS 2D scattering image showing the effect of successive addition of metal salts to silk fabric. The line-collimated primary beam is at the very bottom in these images and different scattering features are highlighted as F1 to F6.

The SAXS data provide insights into the impact of the metal salt incorporation on the nanoscale structure of the silk fibres. 2D SWAXS images of NW and weighted silk samples are shown in Figure 7. 

**Figure 8:**
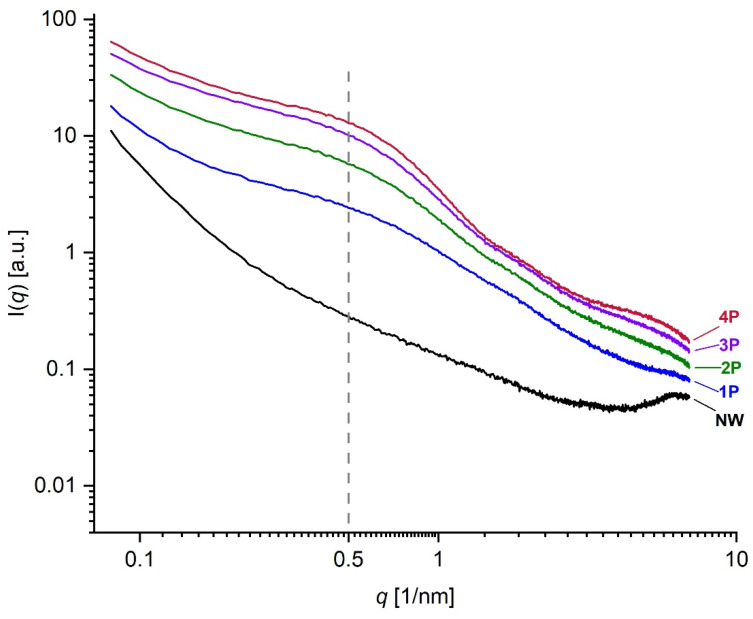
Log-Log SAXS spectra for NW, 1P, 2P, 3P, and 4P samples showing the difference between weighted and NW silk mock-up samples with the increasing concentrations of metal salts. The peak at approximately 0.5 nm^−1^ corresponds to the F1 region in Figure 7.

The NW sample in Figure 7 can be taken as the reference sample and the SWAXS images of the increasingly tin-weighted silk samples follow successively to the right side of the figure. The latter reveals the trend of changes by tin weighting in the typical 2D scattering image. With the accumulated additions of weighting agents, enhanced intensities follow an ascending trend with the increase in metal concentration within the fibres due to tin oxide loading. With the accumulation of tin-weighting salts in the silk from 1P to 4P, one can observe a noticeable successive broadening of the central peak (F1; high-intensity red area that transits to a less intense yellow and green area) around the primary beam, which is positioned at the very bottom of the images. This is the influence of the increasing contents of the tin in silk, which increases the scattering contrast of the fibres and consequently also the scattering intensity. In parallel, this central peak area seems to be increasingly stretching towards larger *q* values. This effect can be monitored in more detail in the SAXS curves in Figure 8, which were measured by the 1D detector and also exhibit a noticeable peak broadening and a scattering intensity increase from the 1P to 4P samples in this regime. Interestingly the peak at around 0.5 nm^−1^ (corresponding to area F1 in Figure 7) seems to more or less keep the position with increasing tin concentration in the samples. According to Bragg’s law, this *q* value corresponds to the distance of about 12.5 nm, proving that tin weighting certainly influences and changes the structure of the silk fibres on the nanoscale size level. 

A second interesting observation is that other scattering peak areas (F2–F5) in Figure 7 do not seem to change considerably for the 1P and 2P samples, which seems to be generally the case even for the 3P and 4P samples. Nevertheless, one could claim a slight broadening of F3–F4 areas at higher tin contents, i.e., 3P and 4P. A significant broadening can be observed just above the F5 area and for the F6 area with tin weighting indicating a noticeable loss of order at the corresponding length scales. Generally, the broadening of the scattering peak in the SWAXS or SAXS curve indicates that the level of order of some structural feature in the sample is reducing or perhaps that the electron density inhomogeneities (arising from some structural correlations in the sample), which cause the individual scattering peak, are becoming less defined or more dispersed in terms of their size or correlation lengths. The sizes of the scattering moieties or the characteristic correlation lengths between the electron density inhomogeneities, namely define the position of the scattering peak in terms of the scattering angle or scattering vector; if they are more polydisperse or disperse, the scattering peaks are correspondingly broader. The fact that F5 and F6 areas are in the range of the scattering vector *q* values from 18 nm^−1^ to 30 nm^−1^ (according to Bragg’s law corresponding to 0.35 nm and 0.21 nm) indicates that tin weighting certainly also affects the structure of the silk fibres close to and on the atomistic-scale size level to some extent [37]. The complete SAXS datasets is available in the Supplementary Materials.

Contact angle analysis revealed the immediate effect on silk surface properties, revealing significant differences in hydrophilicity between NW and weighted silk samples. The complete contact angle measurement data are available in the Supplementary Materials. NW samples showed a gradual decrease in contact angle over time, with initial mean angles of 32.09° to 39.41°, decreasing to 10.32° to 18.51° by the end of measurement. In contrast, 1P and 4P samples exhibited extreme hydrophilicity. Water droplets disappeared within one frame (<0.36 s) for most 1P and 4P samples, preventing quantitative measurements. One 4P sample allowed an initial measurement, showing a mean contact angle of 12.12° before rapid absorption. 

The tin-phosphate-weighting process significantly altered silk surface properties, transforming it from a moderately hydrophobic to a highly hydrophilic material. This change was achieved even with a single weighting treatment. It is important to note that silk hydrophobicity is influenced by several factors, including processing conditions and silk type, resulting in a wide range of hydrophobicities [50,51]. Even though NW samples showed lower hydrophilicity compared to the weighted samples, they are still considered moderately to low hydrophobic within the context of silk materials. 

Overall, these findings highlight the significant impact of tin-phosphate weighting on the silk fabric structure, primarily driven by the incorporation of tin-phosphate nanoparticles throughout the weighting process. These changes indicate that the incorporation of tin-phosphate nanoparticles on and within silk fibres can restrict the flexibility of polymer chains, impacting the physical properties and potentially the degradation behaviour of silk textiles. Therefore, it can be hypothesized that the addition of these nanoparticles alters the mechanical and chemical stability of silk materials, leading to modified performance characteristics and durability.

Additional research is essential to comprehensively elucidate the distinct degradation mechanisms triggered by various environmental stressors acting on weighted and NW silk fibres. Such investigations should encompass the physical, structural, and chemical transformations that silk fibres undergo during the weighting process and subsequent accelerated degradation conditions. A deeper understanding of these phenomena will enable the development of strategies to mitigate deterioration and prolong the longevity of silk-based materials in diverse applications.

## 4. Conclusions

This study employed a multi-technique approach to investigate the structural changes induced in silk fabrics by the tin-weighting process using mock-up samples. Through SEM-EDX, FTIR-ATR, XRD, SAXS, SWAXS, and CA analysis, we have demonstrated that tin weighting significantly alters the nanoscale and molecular structure of silk fibres. Our findings reveal the following:

(1) SEM-EDX analysis showed the deposition of tin-phosphate nanoparticles on the surface of silk fibres and estimated nanofeatures’ average size to be around 20 nm.
(2) FTIR-ATR spectroscopy indicated alterations in the hydrogen bonding network and water accessibility within the silk fibres, while also suggesting a slight increase in β-sheet content in heavily weighted silk compared to NW silk, pointing to some changes in the protein structure.
(3) Using XRD and SWAXS analyses, significant modifications to the crystalline and amorphous regions of silk fibres could be evidenced, with a notable reverse order in crystallinity with a decrease in long-range order and an increase in short-range ordered or amorphous regions.
(4) SAXS analysis further demonstrated changes in the nanoscale structure of silk fibres, particularly at the 12.5 nm scale, in addition to the effect it has on the structure of the silk fibres close to the atomistic-scale size level.
(5) Contact angle measurements demonstrated a significant increase in silk hydrophilicity after tin-phosphate weighting, transforming the material from moderately hydrophobic to highly hydrophilic.
(6) Overall, tin-phosphate weighting significantly alters the microstructure of silk fibres. SEM-EDS showed the nanoscale modification of the fibres by the weighting process. FTIR analysis indicates an increase in β-sheet content, suggesting an enhanced local order within the previously amorphous regions. This is corroborated by XRD data showing increased short-range order, while a decrease in long-range order suggests the formation of smaller, ordered domains. Importantly, the overall fibre structure remains largely intact, as evidenced by SAXS and SWAXS results. A notable consequence of these structural changes is a significant increase in fibre hydrophilicity.
(7) The structural modifications induced by tin-weighting could have important implications for the physical properties and long-term stability of silk textiles. The incorporation of tin-phosphate nanoparticles appears to restrict the flexibility of polymer chains within the silk fibres, potentially affecting their mechanical properties and degradation behaviour.

This study contributes to a deeper understanding of how tin-weighting affects the structure of silk fibres at multiple length scales, paving the way for the development of strategies to mitigate deterioration and prolong the lifetime of silk-based materials in diverse applications, including cultural heritage preservation.

Further research is essential to establish direct correlations between the observed structural changes and specific physical properties of tin-weighted silk fabrics, such as tensile strength, elasticity, and durability. Additionally, accelerated degradation studies under various environmental conditions, the focus of our future work, will provide valuable insights into the impact of these structural modifications on the degradation pathways and longevity of silk textiles.

## Data Availability

The datasets generated and analysed during the current study, including CA, SEM-EDS, XRD, SAXS, and FTIR data, are available in Zenodo at https://doi.org/10.5281/zenodo.13387213.

## Supplementary Materials

The supplementary data for this study are openly available in Zenodo at https://doi.org/10.5281/zenodo.13387213 (accessed on 22 August 2024).
